# *Agaricus blazei* Polysaccharide Alleviates DSS-Induced Colitis in Mice by Modulating Intestinal Barrier and Remodeling Metabolism

**DOI:** 10.3390/nu15234877

**Published:** 2023-11-22

**Authors:** Zhong-Hao Ji, Song He, Wen-Yin Xie, Pei-Sen Zhao, Wen-Zhi Ren, Wei Gao, Bao Yuan

**Affiliations:** 1 Department of Laboratory Animals, College of Animal Sciences, Jilin University, Changchun 130062, China; 2 Department of Basic Medicine, Changzhi Medical College, Changzhi 046000, China

**Keywords:** *Agaricus blazei* polysaccharide (ABP), ulcerative colitis (UC), oxidative stress, gut microbiota

## Abstract

Ulcerative colitis (UC) is a chronic noninfectious intestinal disease that severely affects patients’ quality of life. *Agaricus blazei* Murrill polysaccharide (ABP) is an effective active ingredient extracted from *Agaricus blazei* Murrill (ABM). It has good efficacy in inhibiting tumor cell growth, lowering blood pressure, and improving atherosclerosis. However, its effect on colitis is unclear. The aim of this study was to analyze the protective effects and potential mechanisms of ABP against dextran sulfate sodium (DSS)-induced acute colitis in mice. The results showed that dietary supplementation with ABP significantly alleviated DSS-induced colitis symptoms, inflammatory responses, and oxidative stress. Meanwhile, ABP intervention was able to maintain the integrity of the intestinal mechanical barrier by promoting the expression of ZO-1 and Occludin tight junction proteins and facilitating mucus secretion. Moreover, 16S rRNA sequencing results suggested that ABP intervention was able to alleviate DSS-induced gut microbiota disruption, and nontargeted metabolomics results indicated that ABP was able to remodel metabolism. In conclusion, these results demonstrate that dietary supplementation with ABP alleviated DSS-induced acute colitis by maintaining intestinal barrier integrity and remodeling metabolism. These results improve our understanding of ABP function and provide a theoretical basis for the use of dietary supplementation with ABP for the prevention of ulcerative colitis.

## 1. Introduction

Inflammatory bowel disease (IBD), a group of chronic, noncommunicable gastrointestinal disorders, primarily encompasses ulcerative colitis (UC) and Crohn’s disease (CD) [1]. Characteristic symptoms include abdominal discomfort, diarrhea, and hematochezia, which over an extended period can lead to malnutrition and a substantial reduction in the quality of life for patients [2,3]. Furthermore, IBD may heighten the risk of cardiovascular and neurological diseases, adding to its burden [4,5]. Recently, the global incidence of IBD has been increasing, and it is estimated that by 2030, there will be more than seven million people living with IBD in Europe and the United States. Its prevalence in countries such as those in North America, Oceania, and Europe will exceed 0.3% [6] The etiology of UC remains partially unclear, but indications point toward a complex interplay between genetic factors, environmental influences, and immune system dysregulation [3]. Presently, the primary therapeutic modalities include pharmacological interventions (such as glucocorticoids, immunosuppressants, and biological agents) and surgical procedures, although long-term drug use may yield adverse reactions, posing an additional challenge [7].

Polysaccharides are complex carbohydrates formed by the condensation and dehydration of more than ten monosaccharide molecules [8]. They are one of the fundamental substances that constitute living organisms and are widely present in nature. Research has found that natural polysaccharides exhibit various biological effects, such as antioxidant, anti-tumor, and immunomodulatory activities [9,10,11]. *Ganoderma lucidum* polysaccharide and Huangshan *Floral Mushroom* polysaccharide have been shown to possess prebiotic properties, which can increase the abundance of probiotics and alleviate DSS-induced gut microbiota disorders [12,13]. According to their sources and synthesis, gut microbial metabolites can be divided into three categories: metabolites produced from dietary components by gut bacteria, host-derived metabolites modified by gut bacteria, and metabolites resynthesized by gut bacteria [14,15]. Studies have shown that gut microbial metabolites also play an important regulatory role in the pathogenesis of UC. Polysaccharides have shown some benefits for DSS-induced intestinal metabolic disorders, for example, *Gastrodia elata* polysaccharides are able to regulate metabolic processes, such as tryptophan, cysteine, and vitamin B6 [16]; *Bergamot* polysaccharides have a beneficial effect on tyrosine and phenylalanine metabolism, the synthetic pathways of phenylalanine, tyrosine, and tryptophan organisms are widely regulated, in which the concentration of intestinal L-phenylalanine is significantly increased after Bergamot polysaccharide treatment, and in vitro experiments have shown that L-phenylalanine has anti-inflammatory activity [17]; fucoidan, on the other hand, mainly affects bile acid metabolism, which in turn acts through activation of the Farnesoid X Receptor [18].

*Agaricus blazei* Murrill (ABM) is a medicinal mushroom native to Brazil, whose fruiting bodies and mycelia contain abundant polysaccharides [19,20]. *Agaricus blazei* polysaccharide (ABP) refers to an active component extracted from ABM, primarily consisting of β-glucans [20,21]. Studies have demonstrated that ABP possesses a wide range of biological activities, including antitumor [22,23], antimicrobial [24,25], anti-inflammatory [26,27], antioxidant [28], hypoglycemic, and hypolipidemic effects [29,30]. Li Y et al. [20] reported that ABP had a regulatory effect on dyslipidemia in hyperlipidemic rats, and that the mechanism may be through the modulation of high-fat diet-induced intestinal dysbiosis in rats. Whether ABP is also able to regulate the intestinal flora and intestinal microbial metabolites, thereby influencing the development of UC, has not been reported.

In this study, we assessed the preventive effect of dietary supplementation with ABP using a mouse model of DSS-induced acute colitis and analyzed the effects of ABP on the gut microbiota and metabolic pathways through 16S rRNA sequencing and untargeted metabolomics. The results of the study will deepen our understanding of the function of ABP and lay a theoretical foundation for its further use.

## 2. Materials and Methods

### 2.1. Materials

Dextran sulfate sodium salt (DSS) (molecular weight 36–50 kDa) (MP Biomedicals, Santa Ana, CA, USA) and *Agaricus blazei* polysaccharide (ABP) were purchased from Shanxi Rongling Biotechnology Co. (Xi’an, China). Streptomycin, ampicillin, gentamicin, and vancomycin were purchased from Dalian Meilun Biotechnology Co. (Dalian, China). Anti-Muc2, anti-Claudin 1, anti-ZO-1, and anti-Occludin antibodies were purchased from Affinity Biosciences (Cincinnati, OH, USA). Anti-GAPDH and anti-rabbit IgG antibodies were purchased from Cell Signaling Technology (Danvers, MA, USA).

### 2.2. Animal Experiments

Twenty-four six- to seven-week-old specific pathogen free male BALB/c mice were purchased from Liaoning Changsheng Biotechnology Co., Ltd. (Shenyang, China) (SCXK (Liao) 2020-0001). Twenty-four mice were acclimated for 1 week prior to randomization into 3 groups of 8 mice each: the negative control group (NC), the model group (DSS), and the ABP intervention group (DSS + ABP). This study was approved by the Animal Ethics and Welfare Committee of Jilin University (License No. SY202305009). All experimental procedures were conducted in strict compliance with animal welfare ethics and animal welfare laws and regulations.

**Figure 1:**
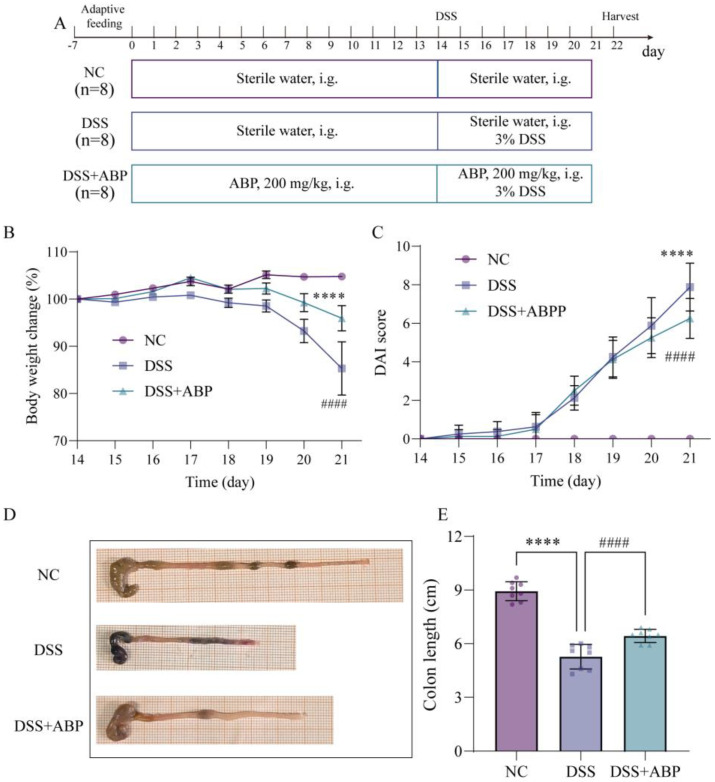
*Agaricus blazei* Murrill polysaccharide (ABP) attenuates dextran sulfate sodium (DSS)-induced colitis symptoms. (**A**) Experimental flow chart; (**B**) the change in body weight in these three groups; (**C**) the disease activity index (DAI) score between these three groups; (**D**) representative images of the colon in the three groups; and (**E**) the colon length in these three groups. Data are presented as mean ± SD, (n = 8). **** *p* < 0.0001; #### *p* < 0.0001 (**** indicates comparison with the NC group and #### indicates comparison with the DSS group).

The experimental design is shown in Figure 1A. During the initial 14 days, all mice received sterile water, whereas the DSS + ABP group received ABP (200 mg/kg, intragastrically (i.g.) (after referencing the literature, 50, 100, and 200 mg/kg/d were utilized in the initial tests, and 200 mg/kg/d was finally selected for the formal experiment)) [18,31,32]. For the next 7 days, both the DSS and DSS + ABP groups were treated with 3% DSS, whereas the DSS + ABP group continued with ABP treatment. The NC group received daily treatment with distilled water. On day 22, the mice were sacrificed under anesthesia for the collection of colon tissues and blood samples.

### 2.3. Disease Activity Index (DAI) Scoring

To assess the disease activity index, body weight, stool consistency, and stool blood were measured and scored following a previously published grading system [33].

### 2.4. Enzyme-Linked Immunosorbent (ELISA) Assay

The colon tissue was added to 9 times the volume of PBS at the ratio of weight (g):volume (mL) = 1:9 and homogenized using a tissue grinder (JingXin, Shanghai, China). Tissue homogenates and blood samples were centrifuged at 4 °C and 3000 rpm for 15 min and then the supernatant was taken for determination. Changes in the levels of inflammatory and oxidation-related factors were detected using a mouse ELISA kit (SINOBESTBIO, Shanghai, China) according to the manufacturer’s protocol. The optical density (OD) was detected with a microplate reader and the concentration of individual samples was calculated.

### 2.5. Tissue Section and Immunohistochemistry (IHC) Staining

The obtained mouse tissue samples were fixed by soaking in 4% paraformaldehyde overnight, embedded in paraffin, and cut into 4 μm thick sections. According to the manufacturer’s protocol, the sections were stained with hematoxylin and eosin (HE), Alcian blue (AB), and periodic acid–Schiff (PAS). The HE staining results were scored in terms of four areas: inflammatory cell infiltration, mucosal edema, crypt swelling disruption, and epithelial cell damage. Positive areas of AB staining and PAS staining were analyzed using the IHC-Toolbox plugin in ImajeJ.

The sections were incubated overnight at 4 °C with a primary antibody against Muc2 (1:200). After washing with PBS, the sections were incubated with HRP antibody at 37 °C for 30 min. Then, the sections were stained with a DAB kit for 10 min and visualized under a light microscope. Positive areas of IHC results were analyzed using the IHC-Toolbox plugin in ImajeJ.

### 2.6. Western Blotting

Tissue proteins were extracted with RIPA buffer and quantified. Samples were separated by SDS‒PAGE and transferred to polyvinylidene fluoride membranes, and the membranes were incubated with the primary antibody (1:1000) overnight at 4 °C. The membranes were washed with Tris-buffered saline containing Tween-20 (TBST) and incubated with HRP-conjugated secondary antibody for 1 h at room temperature. Protein bands were visualized using enhanced chemiluminescence (ECL) substrate and captured on a fully automated chemiluminescence imaging system.

### 2.7. 16S rRNA Sequencing

Microorganisms were collected from mouse fecal samples, and a DNA extraction kit was used to extract DNA according to the manufacturer’s instructions. The extracted DNA was used as a template for PCR amplification of the V3–V4 region using the 338F-806R primer pair. PCR amplification products were gel recovered, purified, and evaluated using an Agilent 2100 Bioanalyzer (Agilent, Santa Clara, CA, USA). Qualified libraries were bipartite sequenced at 2 × 250 bp using an Illumina NovaSeq 6000 sequencer (Illumina, Woburn, MA, USA). Data were filtered and spliced using cutadapt (v1.9), FLASH (v1.2.8), fqtrim, and Vsearch software (v2.3.4). The feature sequence table was obtained after DADA2 noise reduction. Follow-up Bioinformatic analysis was performed using the OmicStudio tools at https://www.omicstudio.cn/ (accessed on 22 September 2023).

### 2.8. Nontargeted Metabolomics Analysis

A total of 100 mg of cecum contents were weighed, ground in liquid nitrogen, and placed in eppendorf tubes. A total of 500 μL of pre-cooled 80% methanol was added and vortexed to mix. The samples were cooled on ice for 5 min and subsequently subjected to centrifugation at 15,000× *g* and 4 °C for a duration of 20 min. A portion of the supernatant was diluted with LC-MS grade water to reach a final concentration of 53% methanol. Next, the samples were transferred to a fresh Eppendorf tube and centrifuged again at 15,000× *g* and 4 °C for 20 min. Finally, a Liquid chromatography tandem mass spectrometry (LC-MS) system analysis was performed by injecting the supernatant. The detection and analysis of non-targeted metabolomics was conducted by Lianchuan Biotechnology (Suzhou, China). A partial least squares discriminant analysis (PLS-DA) was used for supervised classification. Differentially abundant metabolites were identified based on variable importance projection (VIP) [34]. The thresholds for defining differentially abundant metabolites between the groups were as follows: multiplicity of differences ≥1.5 or ≤1/1.5, *p* value < 0.05, and VIP value ≥ 1.

### 2.9. Correlation Analysis

Multiomics data, including 16S rRNA sequencing, metabolomics, and physiological/biochemical indices, were collected. Spearman’s correlation analysis was performed to calculate the correlation coefficients between all the indicators. The ggplot2 and heatmap packages in R were then used to generate visualizations of the correlation matrix and heatmaps.

### 2.10. Statistical Analyses

The experimental data are presented as the mean ± standard deviation (SD). One-way ANOVA followed by Dunnet post-testing was used to compare multiple groups. The data were analyzed and plotted using GraphPad Prism 9.5 (La Jolla, CA, USA). *p* < 0.05 was considered to indicate a significant difference.

## 3. Results

### 3.1. ABP Attenuates DSS-Induced UC in Mice

The in vivo experimental results demonstrated significant differences in multiple parameters between the NC group and the DSS group. Specifically, the DSS group exhibited decreased mouse body weight (*p* < 0.0001) (Figure 1B), elevated DAI scores (*p* < 0.0001) (Figure 1C), and shortened colon length compared to controls (*p* < 0.0001) (Figure 1D,E). Following ABP treatment, all parameters were improved: mouse body weight increased, DAI scores decreased, and colon length increased. These results suggest that ABP has a therapeutic effect in relieving DSS-induced UC, but the intrinsic mechanism through which it occurs needs to be further explored.

### 3.2. ABP Reduces Inflammation and Oxidative Damage

**Figure 2:**
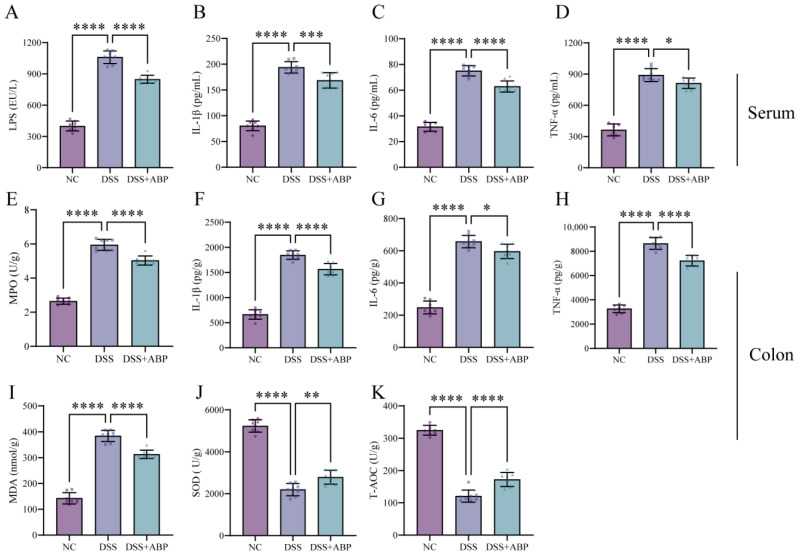
*Agaricus blazei* Murrill polysaccharide (ABP) reduces inflammation and oxidative damage. (**A**–**D**) Cytokine levels in serum for each group. (**A**) Lipopolysaccharide levels; (**B**) IL-1β levels; (**C**) IL-6 levels; and (**D**) TNF-α levels. (**E**–**K**) Colon cytokine levels in each group. (**E**) MDA levels; (**F**) IL-1β levels; (**G**) IL-6 levels; (**H**) TNF-α levels; (**I**) MDA levels; (**J**) SOD levels; and (**K**) T-AOC levels. Data are presented as mean ± SD, (n = 8). * *p* < 0.05, ** *p* < 0.01, *** *p* < 0.001, and **** *p* < 0.0001.

Fruchon S et al. [35,36] reported that ABP is significant in regulating the relationship between inflammatory and oxidative factor expression. ELISA results showed that DSS induction significantly increased the serum levels of LPS, IL-1β, IL-6, and TNF-α in mice compared to the NC group (*p* < 0.0001). However, ABP treatment markedly reduced the expression of these factors (Figure 2A–D). At the same time, the levels of IL-1β, IL-6, and TNF-α in colon tissues were also measured and were consistent with the serum results (Figure 2F–H). DSS was found to increase MPO and MDA expression and decrease SOD and T-AOC levels, whereas ABP reversed these changes (Figure 2E,I–K). These results suggest that ABP intervention can significantly inhibit DSS-induced inflammation and oxidative stress.

### 3.3. ABP Enhanced Intestinal Physical and Chemical Barrier Protection

**Figure 3:**
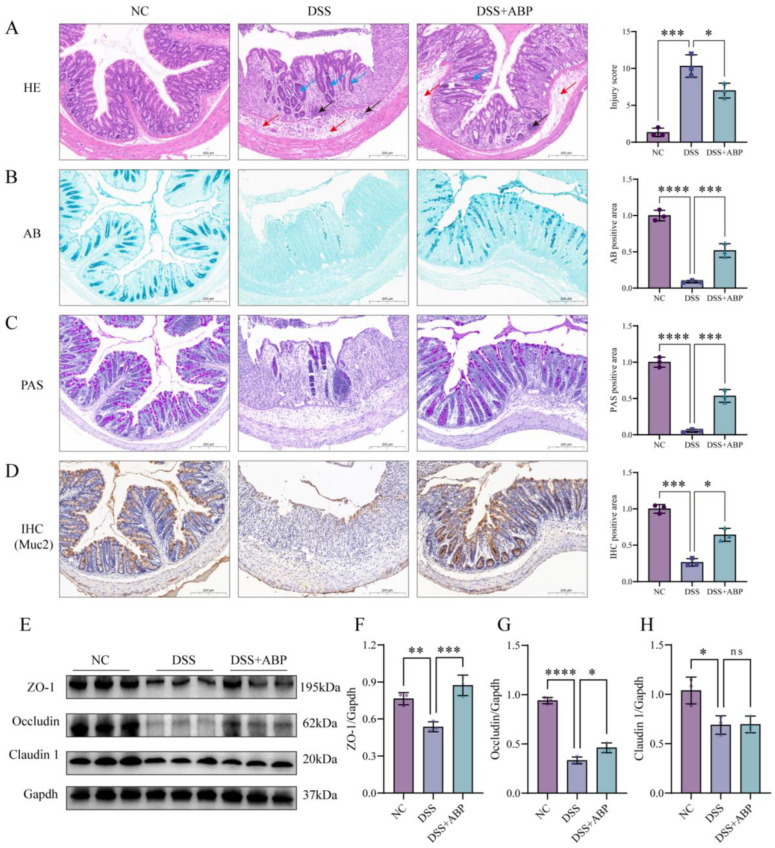
*Agaricus blazei* Murrill polysaccharide (ABP) alleviates dextran sulfate sodium (DSS)-induced intestinal barrier damage. (**A**) Hematoxylin and eosin (HE) staining of colon tissue, red arrows indicate mucosal edema, black arrows indicate inflammatory cell infiltration, and blue arrows indicate crypt damage; (**B**) Alcian blue (AB) staining of colon tissue; (**C**) periodic acid–Schiff (PAS) staining of colon tissue; (**D**) immunohistochemistry detection of Muc2 expression in colonic tissues; (**E**) Western blot strips of tight junction markers; and (**F**–**H**) protein expression analysis of ZO-1, Occludin, and Claudin1. Data are presented as mean ± SD, (n = 3). ns *p* > 0.05, * *p* < 0.05, ** *p* < 0.01, *** *p* < 0.001, and **** *p* < 0.0001.

Colonic histopathological changes and mucus secretion were observed using HE, AB, and PAS staining. The HE staining results showed that DSS treatment disrupted the colonic mucosal structure, with disorganized gland arrangement and disappearance, as well as a decrease in the ratio of villus height to crypt height (Figure 3A). AB staining and PAS staining showed the loss of cup cells and decreased mucus secretion after DSS induction (Figure 3B,C). Immunohistochemistry findings demonstrated markedly down-regulated Muc2 expression in the DSS group (Figure 3D). However, ABP intervention significantly alleviated the DSS-induced changes and repaired colonic mucosal structures, decreased inflammatory cell infiltration, increased mucus secretion, and increased Muc2 expression. In addition, the Western blotting results showed that DSS treatment significantly reduced the levels of ZO-1, Occludin, and Claudin proteins compared to those in the NC group (*p* < 0.05). However, the expression of ZO-1 and Occludin was markedly up-regulated in the ABP treatment group compared to the DSS group (*p* < 0.05), with no significant change in Claudin levels (*p* > 0.05) (Figure 3E–H). These results suggest that ABP is able to alleviate DSS-induced intestinal damage and maintain intestinal barrier integrity.

### 3.4. 16S rRNA Analysis of the Effect of ABP on Gut Microbiota

**Figure 4:**
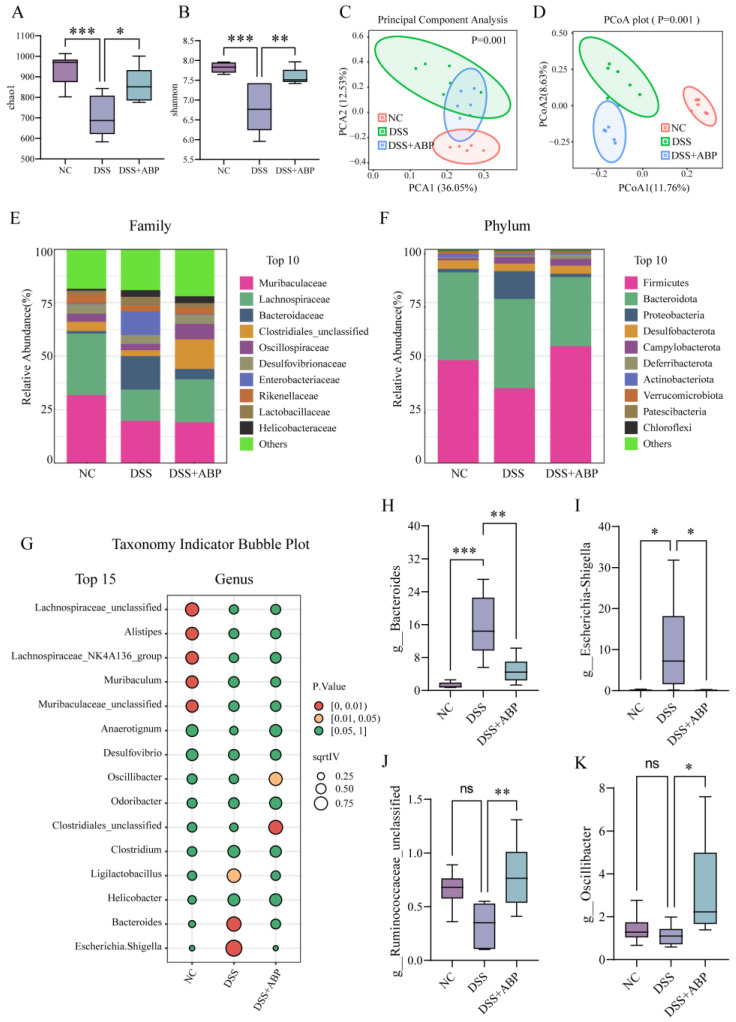
16S rRNA analysis of the effect of *Agaricus blazei* Murrill polysaccharide (ABP) on gut microbiota. (**A**) Chao1 index; (**B**) Shannon index; (**C**,**D**) PCA and PCoA analysis; (**E**,**F**) top 10 display charts of species composition at the family level and phylum level; (**G**) top 15 displays of taxonomy indicators at the genus level; and (**H**–**K**) abundance of four bacterial genera (*Bacteroides*, *Escherichia-Shigella*, *Ruminococcaceae*_unclassified, and *Oscillibacter*) in different groups. Data are presented as mean ± SD, (n = 6). ns *p* > 0.05, * *p* < 0.05, ** *p* < 0.01, and *** *p* < 0.001.

The alpha diversity of the gut microbiota was assessed using the Chao1 and Shannon indices. The results showed that the Chao1 and Shannon indices were significantly lower after DSS treatment (*p* < 0.001), whereas the Chao1 and Shannon indices were significantly higher after ABP intervention (*p* < 0.05) compared to the DSS group (Figure 4A,B). PCA and PCoA analyses were used to assess the beta diversity of gut microorganisms. The results of the downscaling analysis showed that there was a significant separation between the DSS and NC groups, indicating that the composition of their gut microbiota was significantly altered after DSS treatment, whereas the ABP intervention group showed a tendency to regress toward NC (Figure 4C,D). We further analyzed the composition of bacteria at the family and phylum levels among the three groups and identified the top 10 most abundant taxa (Figure 4E,F). However, the ratio of Firmicutes/Bacteroidetes was not significantly different between groups. Indicator species for each group at the genus level are also shown using a bubble chart (Figure 4G). The expression levels of four bacterial genera across three groups at the genus level are also shown. The abundance of *Bacteroides* and *Escherichia-Shigella* was significantly increased (*p* < 0.05), and the abundance of *Ruminococcaceae_unclassified* and *Oscillibacter* did not significantly change (*p* > 0.05) after DSS treatment, whereas the abundance of the above four bacterial genera was significantly higher in the ABP intervention group than in the DSS group (*p* < 0.05) (Figure 4H–K). The above results suggest that ABP intervention can alleviate DSS-induced gut microbiota disruption.

### 3.5. Untargeted Metabolomics Analysis of the Effects of ABP on Metabolites

**Figure 5:**
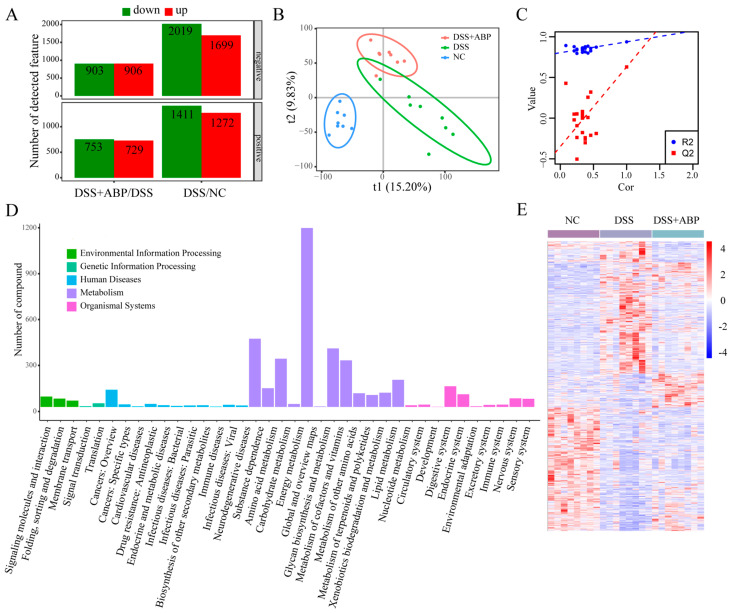
Metabolomics analysis of the effects of *Agaricus blazei* Murrill polysaccharide (ABP) on metabolites. (**A**) Differentially abundant metabolites between groups in positive and negative ionization modes; (**B**) results of PLS−DA analysis; (**C**) replacement test results; (**D**) categorization of differentially abundant metabolites; (**E**) overall metabolite abundance profiles and clustering of groups, (n = 8).

**Figure 6:**
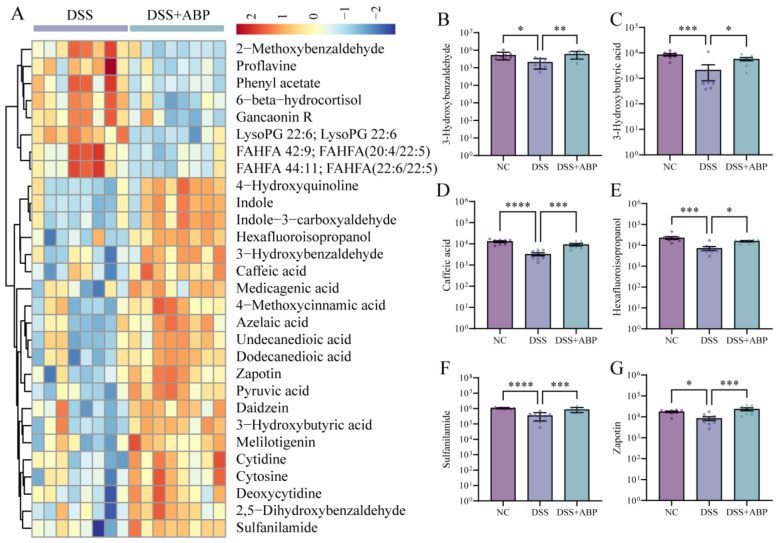
Differentially abundant metabolites. (**A**) Heatmap showing the top 30 metabolites in the order of fold change with a VIP value >1.5; and (**B**–**G**) the abundance of six metabolites (3-hydroxybenzaldehyde, 3-hydrpxubutyric acid, caffeic acid, hexafluoroisopropanol, sulfanilamide, and zapotin) in different groups. Data are presented as mean ± SD, (n = 8). * *p* < 0.05, ** *p* < 0.01, *** *p* < 0.001, and **** *p* < 0.0001.

To further elucidate the effects of ABP in mice, we analyzed the metabolic composition of cecum contents in three groups of mice using untargeted metabolomics. With the threshold of fold change ≥1.5 or ≤2/3, *p* < 0.05, and a VIP value ≥1, in the negative ion mode, there were 2019 metabolites down-regulated and 1699 metabolites up-regulated in the DSS group compared with the NC group. A total of 903 metabolites were down-regulated and 906 metabolites were up-regulated in the DSS + ABP group compared with the DSS group. In the positive ion mode, 1411 metabolites were down-regulated and 1272 metabolites were up-regulated in the DSS group compared with the NC group. A total of 753 metabolites were down-regulated and 729 metabolites were up-regulated in the DSS + ABP group compared with the DSS group (Figure 5A). The results of the PLS-DA analysis suggest that the three groups of samples had different metabolic profiles, and the permutation test results suggest that the PLS-DA downscaling model was not overfitted (Figure 5B,C). The classification of differentially abundant metabolites in each group is shown in Figure 5D, and the overall metabolic profiles and clustering of the three groups are shown in Figure 5E. A heatmap showing the top 30 metabolites in the order of fold change with a VIP value >1.5 is shown in Figure 6A. Among them, the levels of tryptophan metabolites Indole and Indole-3-carboxyaldehyde were significantly higher in the intestine after ABP intervention compared to the DSS group (Figure 6A). Further, the contents of six metabolites (3-hydroxybenzaldehyde, 3-hydrpxubutyric acid, caffeic acid, hexafluoroisopropanol, sulfanilamide, and zapotin) in the intestinal contents of the three groups of mice were shown in the form of bar graphs. The results showed that the content of the six metabolites was significantly reduced (*p* < 0.05) after DSS induction, whereas the intervention of ABP resulted in a significant recovery of the content of these six metabolites (*p* < 0.05) (Figure 6B–G). The above results suggest that ABP can remodel DSS-induced metabolic disorders.

### 3.6. Correlation Analysis of Differential Bacteria, Differentially Abundant Metabolites, and Physiological and Biochemical Indicators

**Figure 7:**
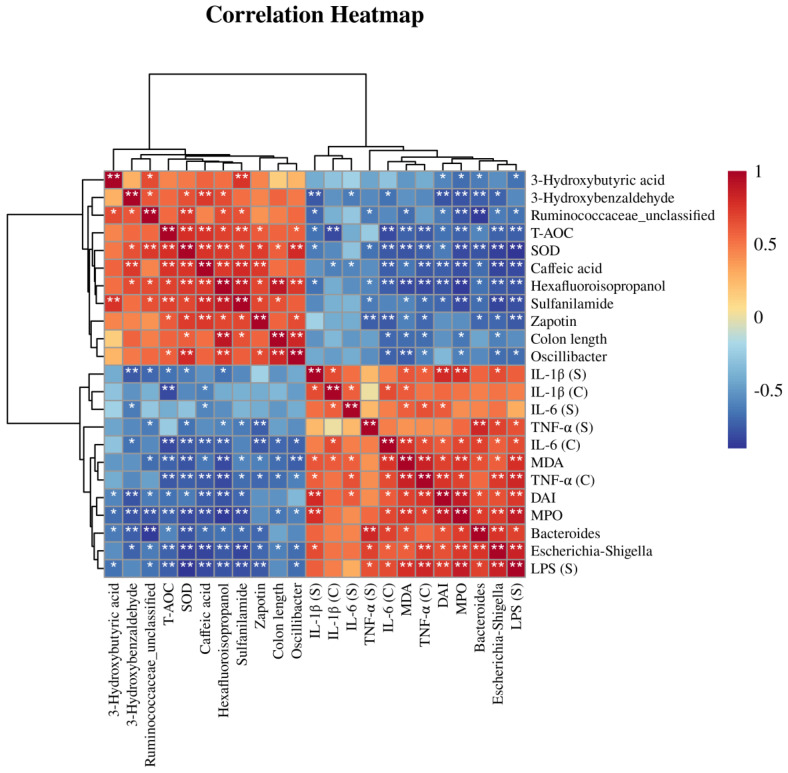
Spearman correlation analysis. A heatmap construction for the correlation analysis of gut microbiota, metabolites, and physiological and biochemical indicators. White star indicates significance analysis, * *p* < 0.05 and ** *p* < 0.01.

We performed a correlation analysis on the four key bacteria, the six differentially abundant metabolites, and the physiological biochemical indicators previously detected. A heatmap was used to demonstrate the correlation relationships among these factors (Figure 7). The correlation analysis showed that *Bacteroides* and *Escherichia-Shigella* were positively correlated with the levels of pro-inflammatory factors, pro-oxidant factors, and DAI scores, and negatively correlated with the levels of antioxidant factors and the six metabolites. *Ruminococcaceae*_unclassified and *Oscillibacter* were negatively correlated with pro-inflammatory factor levels, pro-oxidant factor levels, and DAI scores, and positively correlated with antioxidant factors and the levels of the six metabolites. The correlation heatmap and network diagram provided insights into the mechanism through which ABP alleviates DSS-induced colitis, but the underlying mechanisms need to be further verified with additional studies.

## 4. Discussion

ABP has potent antitumor effects through regulating immune cell differentiation and/or function, inhibiting proliferation, and inducing apoptosis in cancer cells [37,38,39]. Additionally, ABP exerts anti-inflammatory, antioxidant, and hypoglycemic activities by reducing oxidative stress and blood glucose and lipids levels [40,41,42]. In the present study, we evaluated the protective effect of dietary supplementation with ABP using a mouse model of DSS-induced acute colitis. The results showed that ABP intervention significantly improved DSS-induced colitis symptoms. It has been reported that damage to the intestinal epithelial barrier can increase the permeability and infiltration of pathogens, induce barrier dysfunction, and manifest as cellular apoptosis, erosion, and ulceration, representing a key pathological change in UC [43,44]. Epithelial tight junctions are key structures that regulate the transport of macromolecules across cells. Dysregulation of the expression of tight-junction-related proteins is closely related to UC progression [45,46]. In addition, inflammation-related cytokines can influence the expression of tight junction proteins and change the permeability of the intestinal epithelium by affecting tight junction proteins, thereby participating in and altering UC developmental processes [47,48]. Therefore, we further explored the expression levels of inflammatory and oxidative factors, as well as three tight junction proteins (ZO-1, Occludin, and Claudin1).

In the DSS-induced UC model, we clearly observed significant up-regulation in the expression of inflammatory factors, such as TNF-α and IL-1β, in serum and colon tissues. Meanwhile, the significant increase in MPO and MDA oxidative stress markers and the significant decrease in SOD and T-AOC antioxidant markers were consistent with the expected outcomes. ABP exhibited excellent counteracting effects on DSS-induced inflammation and oxidative responses, aligning with previous reports [49,50,51]. In this experiment, DSS induced damage to the intestinal barrier in mice, and the expression levels of three tight junction proteins, ZO-1, Occludin, and Claudin1, were significantly reduced, as evidenced by mucosal disruption, ulceration, and the disappearance of glands in the tissue sections. The expression levels of tight junction proteins were increased, and damage to the intestinal barrier was reduced by ABP treatment. Therefore, in this DSS-induced UC mouse model, ABP treatment attenuated DSS-induced intestinal inflammation and oxidative stress, thereby protecting intestinal barrier function possibly through up-regulating tight junction protein expression.

Existing research provides evidence that gut microbiota dysbiosis, intestinal hypersensitivity, and aberrant mucosal immune responses collectively contribute to UC development. A complex interplay between perturbations in these components is believed to drive uncontrolled intestinal inflammation in genetically susceptible individuals [52,53]. In addition, gut microbiota dysbiosis can also trigger inflammatory spillover to other organs, such as joints, the oral cavity, and eyes, leading to inflammation or even cancer [54]. We performed 16S rRNA sequencing to analyze the changes in the gut microbiota of mice. The top 15 genera with the most significant differential expression were selected from the sequencing results. As shown in Figure 4G, the levels of *Ligilactobacillus*, *Bacteroides*, and *Escherichia-Shigella* were markedly elevated in the DSS group. Four bacterial strains were subsequently screened for further detection and validation.

*Bacteroides* are a type of gut microbiota that play crucial roles in human gut nutrition, metabolism, immune response, and overall health [55]. Dysbiosis of *Bacteroides* has been associated with various gastrointestinal disorders, including UC, and metabolic diseases, such as diabetes and obesity [56,57,58]. *Escherichia* and *Shigella* are both Gram-negative bacilli that commonly inhabit the gastrointestinal tract as commensal organisms in the human gut [59]. However, under certain conditions, virulent strains have the potential to breach the intestinal epithelium and induce intestinal disorders [60,61]. It has also been reported that *Ruminococcaceae*_unclassified, as part of the gut microbiota, possesses strong metabolic capabilities for carbohydrates and can perform normal food digestion and absorption functions in the gut [62]. *Oscillibacter* is able to maintain gut pH balance by producing substances such as propionate and butyrate, thus promoting the health and stability of the intestinal mucosa [63,64]. Therefore, these two types of bacteria are generally considered beneficial, but their imbalance or depletion has been closely associated with inflammatory bowel disease, obesity, and other related conditions [65]. The results of the present study showed that the relative abundance of *Lactobacillus* and *Shigella* was significantly increased in DSS-induced UC, which is also consistent with the findings of Schirmer M [66]. Interestingly, treatment with ABP was effective in alleviating DSS-induced intestinal dysbiosis because the levels of *Bacteroides* and *Escherichia-Shigella* were significantly reduced after treatment, and the relative abundance of *Ruminococcaceae*_unclassified and *Oscillibacter* was significantly increased. The increase in these beneficial bacteria leads to improved preventive and protective effects on the intestinal tract, which is also the role of ABP.

Changes in the gut microbiota affect the composition of gut metabolites, including short-chain fatty acids, amino acid metabolites, bioactive peptides, and vitamins. These metabolites have the ability to regulate the immune system, influence energy metabolism, and modulate the intestinal mucosal barrier function [67,68,69]. Thus, the gut microbiota and gut metabolites interact closely with each other and together influence the health and disease states of the body.

In order to assess the effect of ABP intervention on gut metabolism, we performed an untargeted metabolomic analysis of cecum contents based on LC-MS technology. The results showed that the abundance of several metabolites, including 4-hydroxyquinoline, was up-regulated or decreased in DSS-induced UC mice after ABP treatment. The expression levels of 3-hydroxybenzaldehyde, 3-hydroxybutyric acid, caffeic acid, and zapotin have been reported to be closely associated with the inflammatory response and oxidative stress and have therapeutic potential for UC [70,71,72,73]. Subsequently, we examined the abundance of six metabolites (3-hydroxybenzaldehyde, 3-hydroxybutyric acid, caffeic acid, hexafluoroisopropanol, sulfanilamide, and zapotin) identified in the sequencing analysis. As expected, the abundance of these metabolites significantly decreased in DSS-induced UC and significantly increased after ABP treatment.

In summary, we discovered that ABP is able to reduce inflammation and oxidative stress, as well as repair damaged gut barriers, and may also exert its effects through modulation of the gut microbiota and metabolites.

However, our experimental results have certain limitations. Although our study demonstrated significant improvement of DSS-induced UC by ABP, as validated by multidimensional analysis, the recommended intake and potential toxic side effects of ABP in human subjects remain unknown. Furthermore, this study did not directly explore and validate the key bacterial and metabolite changes in the gut microbiota during the treatment of UC with ABP, as well as their respective regulatory roles. These aspects require further investigation. Nevertheless, it is undeniable that our study enhances our understanding of the functionality of ABP and provides a theoretical basis for the use of ABP dietary supplements in the prevention of UC.

## Data Availability

Data will be made available on request.
